# A Unique Co-culture Model for Fundamental and Applied Studies of Human Fetoplacental Steroidogenesis and Interference by Environmental Chemicals

**DOI:** 10.1289/ehp.1307518

**Published:** 2014-01-31

**Authors:** Andrée-Anne Hudon Thibeault, Kathy Deroy, Cathy Vaillancourt, J. Thomas Sanderson

**Affiliations:** INRS-Institut Armand-Frappier and BioMed Research Centre, Université du Québec, Laval, Québec, Canada

## Abstract

Background: Experimental tools for studying the complex steroidogenic interactions that occur between placenta and fetus during human pregnancy are extremely limited.

Objectives: We aimed to develop a co-culture model to study steroidogenesis by the human fetoplacental unit and its disruption by exposure to environmental contaminants.

Methods: We cultured BeWo human choriocarcinoma cells, representing the villous cytotrophoblast, and H295R human adrenocortical carcinoma cells, representing the fetal unit, in a carefully adapted co-culture medium. We placed H295R cells in 24-well plates and BeWo cells on transwell inserts with or without pesticide treatment (atrazine or prochloraz) and assessed CYP19 activity and hormonal production after 24 hr of co-culture.

Results: The co-culture exhibited the steroidogenic profile of the fetoplacental unit, allowing a synergistic production of estradiol and estriol (but not of estrone) of 133.3 ± 11.3 pg/mL and 440.8 ± 44.0 pg/mL, respectively. Atrazine and prochloraz had cell-type specific effects on CYP19 activity and estrogen production in co-culture. Atrazine induced CYP19 activity and estrogen production in H295R cells only, but did not affect overall estrogen production in co-culture, whereas prochloraz inhibited CYP19 activity exclusively in BeWo cells and reduced estrogen production in co-culture by almost 90%. In contrast, prochloraz did not affect estradiol or estrone production in BeWo cells in monoculture. These differential effects underline the relevance of our co-culture approach to model fetoplacental steroidogenesis.

Conclusions: The co-culture of H295R and BeWo cells creates a unique *in vitro* model to reproduce the steroidogenic cooperation between fetus and placenta during pregnancy and can be used to study the endocrine-disrupting effects of environmental chemicals.

Citation: Hudon Thibeault AA, Deroy K, Vaillancourt C, Sanderson JT. 2014. A unique co-culture model for fundamental and applied studies of human fetoplacental steroidogenesis and interference by environmental chemicals. Environ Health Perspect 122:371–377; http://dx.doi.org/10.1289/ehp.1307518

## Introduction

Appropriate fetoplacental communication is required for healthy pregnancy, and sex steroid hormones play an essential role in maintenance of pregnancy and fetal development. Pregnant women are exposed to various potential endocrine-disrupting chemicals through diet, medication use, occupational or environmental activities, and other lifestyle factors (Myllynen et al. 2005). Risks posed by chemical exposures are a focus of the Inter-Organization Programme for the Sound Management of Chemicals as stated in its 2012 report (World Health Organization/United Nations Environment Programme 2013). Most of these chemicals will pass through the placental barrier and enter the placenta and fetus, as evident from the presence of contaminants in placental tissues, amniotic fluid, and/or fetal blood (Foster et al. 2000; Ikezuki et al. 2002; Leino et al. 2013). Epidemiological studies have associated altered pregnancy and fetal outcomes with exposure to contaminants such as heavy metals, polychlorinated biphenyls, dioxins, and pesticides (Siddiqui et al. 2003; Stasenko et al. 2010; Weselak et al. 2008). Adverse effects include reduced birth weight, altered fetal cognitive and reproductive tract development, and increased risk of premature birth and spontaneous abortion. Some of these adverse effects may be a consequence of chemical-induced alterations in estrogen production by the syncytiotrophoblast, the functional endocrine unit of the placenta (Albrecht and Pepe 1999; Siddiqui et al. 2003; Stasenko et al. 2010; Weselak et al. 2008). Several processes regulated by estrogens, such as uteroplacental blood flow, trophoblast invasion, and syncytialization are necessary for healthy pregnancy (Albrecht and Pepe 1999; Cronier et al. 1999; Yashwanth et al. 2006). Disruptions of these functions are associated with serious obstetric complications, including altered fetal development, preterm birth, preeclampsia, and intrauterine growth restriction (Albrecht et al. 2005; Kaufmann et al. 2003). The importance of regulation of local estrogen levels during pregnancy was notably underlined by toxicological studies of the well-known estrogenic compound diethylstilbestrol (DES). Exposure *in utero* to DES resulted in severe malformations and malfunctioning of male and female reproductive organs (Norgil Damgaard et al. 2002; Toppari et al. 2010).

Crucially, the human placenta is not in itself capable of producing androgens *de novo* because it lacks significant steroid 17α-hydroxylase/17,20-lyase activity catalyzed by the cytochrome P450 enzyme CYP17 (Braunstein 2003). Therefore, estrogen production by the trophoblast relies on sufficient quantities of fetal and maternal androgen precursors (Rainey et al. 2004), which act as substrates for placental aromatase (CYP19). Among estrogens, estriol, which is uniquely produced by the fetoplacental unit, predominates during pregnancy and is used as a diagnostic marker of fetal well-being (Mucci et al. 2003). Thus, a finely tuned cooperation between placenta and fetus is essential for a healthy pregnancy.

Unfortunately, experimental tools for studying the complex steroidogenic interactions that occur during human pregnancy are extremely limited. Invasive experimental approaches using humans are not possible for obvious ethical reasons. Although *in vivo* rodent models may be useful for specific gene inactivation studies (Stokes 2004), human steroidogenesis during pregnancy differs vastly, making rodent models irrelevant for human studies. In contrast to human pregnancy, the rodent placenta does not synthesize estrogens because it does not express CYP19 or display aromatase activity (Malassine et al. 2003). *In vitro* models have been used to assess hormonal secretion from placenta or fetal cells, but they can provide only partial information because they do not take into consideration the steroidogenic interactions between placenta and fetus. To study those interactions, we developed an *in vitro* co-culture model using H295R human adrenocortical carcinoma (fetal compartment) and BeWo human choriocarcinoma (villous trophoblast compartment) cells. H295R cells possess all the enzymatic capacities of the undifferentiated or fetal-like adrenal gland (Gazdar et al. 1990; Montanaro et al. 2005; Sanderson 2009; Staels et al. 1993) and they produce 16α-hydroxylated androgens (Gazdar et al. 1990), suggesting they can provide the uniquely fetal precursors for the human pregnancy estrogen, estriol. BeWo cells have a high degree of similarity to the villous trophoblast and can, for example, be induced to fuse and form syncytiotrophoblasts that behave like the human syncytium (Nampoothiri et al. 2007). Also, as in syncytiotrophoblasts, basal CYP19 activity in BeWo cells is relatively high. We evaluated the co-culture of H295R and BeWo cells as a model of steroidogenesis and, specifically, of estrogen production in the fetoplacental unit and its disruption by chemical exposures.

## Materials and Methods

*Cells and co-culture conditions*. We cultured BeWo human placental choriocarcinoma cells [catalog no. CCL-98; ATCC, Manasses, VA, USA) in Dulbecco’s modiﬁed Eagle’s medium (DMEM)/F-12 without phenol red, supplemented with 0.6 g/L sodium bicarbonate (NaHCO_3_) (Sigma-Aldrich, Oakville, Ontario, Canada) and 10% fetal bovine serum (FBS; Hyclone, Tempe, AZ, USA). We cultured H295R human adrenocortical carcinoma cells (catalog no. CRL-2128; ATCC) in DMEM/F-12 without phenol red, supplemented with 1.2 g/L NaHCO_3_ (Sigma-Aldrich), 2.5% NuSerum (BD Biosciences, Mississauga, Ontario, Canada), 2 mg/L pyridoxine⋅HCl (Sigma-Aldrich), and 1% ITS + Premix (BD Biosciences). Experiments were performed using cells between passages 7 and 25.

We cultured cells in 75-cm^2^ filter-cap culture flasks (Techno Plastic Products, MIDSCI, St. Louis, MO, USA) in a humidified atmosphere containing 5% carbon dioxide (CO_2_) at 37°C. At 90% confluence, cells were trypsinized [0.5% trypsin (Sigma-Aldrich)] and transferred to new 75-cm^2^ flasks. We added suspensions of H295R cells (2.5 × 10^4^ cells/well) to the wells of one set of 24-well plates, and we added BeWo cells (1.25 × 10^4^ cells/insert) to transwell (Corning Life Sciences, Corning, NY, USA) clear polycarbonate membrane inserts with 0.4-μm pores of another set of 24-well plates, with each cell type in its respective regular growth medium. We removed the regular media 24 hr after seeding, assembled the co-culture (placing inserts with BeWo cells into the wells with H295R cells), and added co-culture medium (0.8 mL/well; 0.2 mL/insert). The co-culture medium was based on ATCC-recommended H295R medium but was supplemented with 1% stripped FBS. For full protocol, see Supplemental Material, Figure S1.

*Chemicals*. Phorbol-12-myristate-13-acetate (PMA), forskolin, formestane, atrazine, and prochloraz were obtained from Sigma-Aldrich. PMA and forskolin are inducers of CYP19 via protein kinase C (PKC) and protein kinase A (PKA) pathways, respectively; formestane is an irreversible inhibitor of CYP19. We dissolved each compound in DMSO to make 1,000-fold concentrated stock solutions. We exposed the cells to various concentrations of each compound in culture medium with a final DMSO concentration of 0.1%. Inserts and wells always contained treated medium from the same solution.

*Cell proliferation*. We monitored cell proliferation quantitatively and in real time in a humidified atmosphere with 5% CO_2_ at 37°C using an xCELLigence™ RTCA DP instrument (ACEA Biosciences, San Diego, CA, USA). This instrument measures changes in impedance detected by gold electrode microarrays at the bottom of each well of a 16-well E-plate (ACEA Biosciences) to which the cells are attached. Before each experiment, we corrected cell impedance for background signals, which corresponded to the cell index measured after equilibrating the E-plate for 30 min with 100 μL appropriate culture medium. We added BeWo and H295R cells to 16-well E-plates in 100 μL at optimized densities of 1 × 10^4^ and 2 × 10^4^ cells/well, respectively. We normalized cell index after cell adherence, which took 3 hr for H295R and 6 hr for BeWo cells. In co-culture experiments, we seeded cells in co-culture medium in E-plates or in E-plate inserts with 0.4 μm pores (ACEA Biosciences) at the above-mentioned cell densities. We assembled the co-culture 24 hr later and refreshed the co-culture medium with the treatments (130 μL/well; 70 μL/insert) (for details, see Supplemental Material, Figure S1). Using ACEA Biosciences RTCA software, version 1.2.1 (http://www.aceabio.com/product_info.aspx?id=187), we collected cell impedance data every 10 min to calculate doubling times from the slope of the linear phase of the proliferation curves.

*CYP19 catalytic activity*. We determined CYP19 catalytic activity by tritiated water-release assay according to the method of Lephart and Simpson (1991) adapted by our laboratory (Sanderson et al. 2000). Briefly, we cultured BeWo (2.5 × 10^4^ cells/well) or H295R (5 × 10^4^ cells/well) cells in 24-well plates in their regular media or in co-culture medium for 24 hr. Cells were then exposed to 54 nM 1β-^3^H-androstenedione (PerkinElmer, Wellesley, MA, USA) in serum-free culture medium for 1.5 hr at 37°C. The conversion of substrate was linear over this time. For co-culture experiments, we assembled and treated the co-culture as described above (see Supplemental Material, Figure S1). Then, we separated the inserts from the wells and placed them directly in the bottoms of the wells of a 12-well plate. We measured CYP19 activity in the wells and inserts separately. We preserved the culture media (insert and well were pooled) at –80°C for subsequent analysis of hormone production.

*Hormone quantification*. We determined hormone production by ELISA using assay kits from DRG Diagnostics (Marburg, Germany) and Abnova (Taipei City, Taiwan) (for details, see Supplemental Material, Table S1).

*Statistical analysis*. We performed experiments at least three times using different cell passages; treatments were performed in triplicate per experiment. We determined statistically significant (*p* < 0.05) differences by two-way analysis of variance (ANOVA) followed by a Bonferroni post hoc test or one-way ANOVA followed by a Tukey post hoc test or Student’s *t*-test, depending on the experimental design, using GraphPad Prism (version 5.04; GraphPad Software, San Diego, CA, USA).

## Results

*Characterization of each individual cell-type under co-culture conditions*. The co-culture medium did not alter the proliferation rate of either cell type compared with those in their regular recommended media (see Supplemental Material, Figure S2A,B). However, we observed that after plating, regardless of the culture medium, BeWo cells required an adaptation period before proliferating, whereas H295R cells proliferated without delay (see Supplemental Material, Figure S2A,B). Doubling times determined from the linear sections (24–72 hr) of the proliferation curves were not significantly different whether H295R or BeWo cells were grown in co-culture medium (35.9 ± 2.3 hr and 25.2 ± 3.6 hr, respectively) or their respective regular media (30.9 ± 1.9 hr and 22.9 ± 2.7 hr, respectively). When placed in co-culture with BeWo cells, H295R cell proliferation over a period of 72 hr was reduced, although this effect was not observed until > 24 hr of co-culture (see Supplemental Material, Figure S2C). When cultured together for < 24 hr, H295R cell doubling time (39.3 ± 6.1 hr) in the presence of BeWo cells was not significantly different from that of cells in monoculture using co-culture medium (34.1 ± 5.3 hr). In contrast, BeWo cell proliferation was not affected by the presence of H295R cells in co-culture (see Supplemental Material, Figure S2D).

Under our co-culture conditions, basal CYP19 activity in BeWo cells (32.5 ± 7.0 fmol/hr) was about 15 times greater than that in H295R cells (2.2 ± 0.4 fmol/hr) and CYP19 activity was unaffected by the presence of the other cell type (data not shown). In each cell line, PMA and forskolin induced CYP19 activity, although induction was more pronounced in H295R than BeWo cells (see Supplemental Material, Figure S3). CYP19 activity and its inducibility were not different in either H295R or BeWo cells whether we cultured the cells in their respective regular media or in the co-culture medium (see Supplemental Material, Figure S3).

Basal production of β-human chorionic gonadotropin (β-hCG), a biochemical indicator of trophoblast health, was 7.7 ± 1.8, 40.5 ± 9.3, and 89.1 ± 14.1 mIU for BeWo cells after 24, 48, and 72 hr in regular medium; in the co-culture medium, it was 10.9 ± 3.2, 80.5 ± 17.8, and 88.5 ± 19.6 mIU (see Supplemental Material, Table S2). Forskolin, a known stimulant of the fusion and biochemical differentiation of BeWo cells, increased β-hCG production markedly. After 48 hr, basal and forskolin-induced β-hCG production were greater in co-culture medium than in regular medium (see Supplemental Material, Table S2), although this increase was not observed after 24 or 72 hr of culture. Basal and forskolin-stimulated β-hCG production by BeWo cells (over a 24-hr period) was not affected when co-cultured with H295R cells (see Supplemental Material, Table S2).

**Figure 1:**
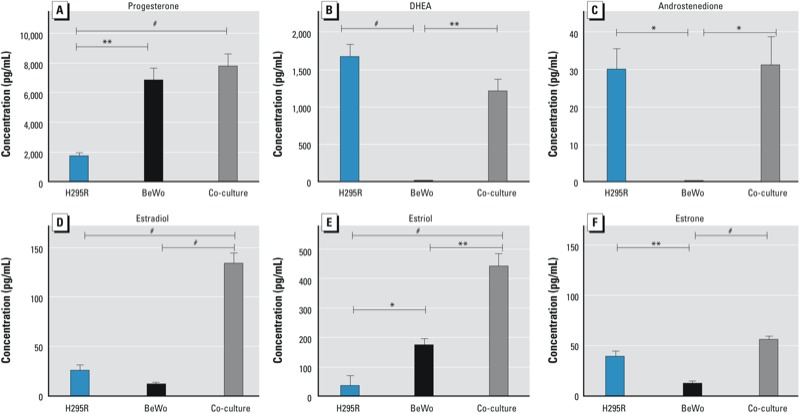
Progesterone (*A*), DHEA (*B*), androstenedione (*C*), estradiol (*D*), estriol (*E*), and estrone (*F*) production by H295R and BeWo cells in monoculture or in co-culture over a 24-hr period. Hormone concentrations (pg/mL) are presented as mean ± SE (progesterone, *n* = 4; DHEA, androstenedione, estriol, and estrone, *n* = 3; estradiol, *n* = 5). *p < 0.05. **p < 0.01. ^#^*p* < 0.001.

*Steroidogenesis in the H295R/BeWo co-culture model*. BeWo cells, representing the placental compartment, mainly produced progesterone after 24 hr in co-culture (Figure 1A); whereas H295R cells, representing the fetal compartment, exclusively produced dehydroepiandrosterone (DHEA) and androstenedione (Figure 1B,C). Testosterone production was not detected (see Supplemental Material, Table S1). Basal estrogen production over a 24 hr period (Figure 1D–F) was relatively low in H295R and BeWo cells in monoculture, with estriol production being greater in BeWo cells than in H295R cells (173 ± 22 vs. 35 ± 35 pg/mL), whereas the opposite was seen for estrone (11.7 ± 3.2 vs. 34.7 ± 1.7 pg/mL). The production of estradiol (133 ± 11 pg/mL) and estriol (441 ± 44 pg/mL) increased synergistically when we placed the two cell types in co-culture (Figure 1D,E), whereas the increase in estrone production (55.1 ± 3.9 pg/mL) was additive. Estrogen production was not saturated because PMA- and forskolin-treated cells in co-culture produced estradiol levels of 422 ± 137 and 954 ± 264 pg/mL, respectively (data not shown).

**Figure 2:**
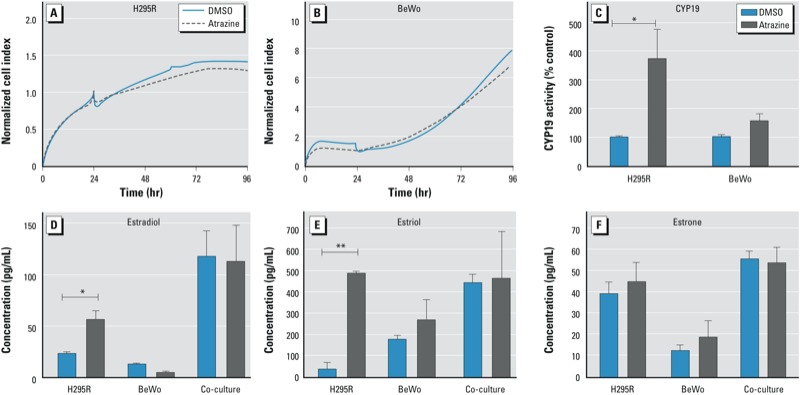
Effects of atrazine (30 μM) on the proliferation of H295R (*A*) and BeWo (*B*) cells in co-culture monitored in real time and its effects on CYP19 activity in each cell line after 24 hr of co-culture (*C*). The effects of atrazine on estradiol (*D*), estriol (*E*), and estrone (*F*) production by H295R and BeWo cells in monoculture or co-culture (24-hr exposure). Concentrations are presented as mean ± SE; *n* = 3. *p < 0.05,**p < 0.01, compared with DMSO control.

**Figure 3:**
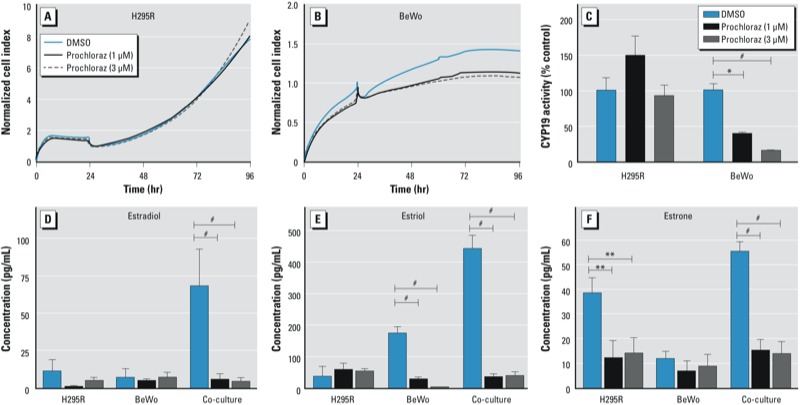
Effects of prochloraz (1 and 3 μM) on the proliferation of H295R (*A*) and BeWo (*B*) cells in co-culture monitored in real time and its effects on CYP19 activity in each cell line after 24 hr of co-culture (*C*). The effects of prochloraz on estradiol (*D*), estriol (*E*), and estrone (*F*) production by H295R and BeWo cells in monoculture or co-culture (24-hr exposure). Concentrations are presented as mean ± SE; *n* = 3. *p < 0.05, **p < 0.01, and ^#^*p* < 0.001, compared with DMSO control.

*Effects of atrazine and prochloraz in the co-culture model*. Treatment with atrazine (30 μM) did not alter the proliferation rate of either cell type in co-culture over a 72-hr period compared with vehicle control (Figure 2A, 2B). However, atrazine, after a 24-hr exposure, induced CYP19 activity to a statistically significant extent in H295R, but not in BeWo cells (Figure 2C). The 3-fold greater CYP19 activity that atrazine produced in the H295R compartment did not result in an increased production of estradiol, estriol, or estrone by the co-culture (112 ± 30, 459 ± 224, and 53.5 ± 7.4 pg/mL, respectively) (Figure 2D–F). Treatment with prochloraz (1 and 3 μM) did not alter the proliferation rate of either cell type in co-culture during the first 24 hr (Figure 3A,B). However, during this period, prochloraz decreased CYP19 activity concentration-dependently in BeWo, but not in H295R cells (Figure 3C). The 2.5- and 6.8-fold inhibition (to 39% and 15% of control, respectively) of CYP19 activity in BeWo cells by 1 and 3 μM prochloraz, respectively, translated into a > 90% inhibition of estradiol and estriol, and 80% inhibition of estrone production by the co-culture, with 1 μM prochloraz reducing estradiol, estriol, and estrone concentrations to 5.3 ± 4.3, 34.6 ± 9.3, and 15.0 ± 4.5 pg/mL, respectively; and 3 μM prochloraz, to 4.1 ± 2.8, 38.2 ± 11.8, and 13.9 ± 4.9 pg/mL, respectively. (Figure 3D–F). Neither atrazine nor prochloraz affected the production of β-hCG in co-culture after a 24-hr exposure (data not shown).

## Discussion

*Establishing the H295R/BeWo co-culture model*. We have succeeded in developing a co-culture of H295R human adrenocortical carcinoma cells with characteristics of the fetal adrenal and BeWo human choriocarcinoma cells with characteristics of the villous trophoblast that exhibits the steroidogenic functionality of the human fetoplacental unit.

A major challenge in the development of our co-culture model was to maintain the unique characteristic of each individual cell line in the co-culture medium, which we adapted to accommodate the culture requirements of both cell lines. In the co-culture medium, the concentration of FBS, which is required for BeWo cells, was reduced to 1% and was charcoal-stripped to remove steroids and limit interference with endogenous steroid hormone production by the co-culture. H295R cells do not tolerate high concentrations of FBS but were not affected by the presence of 1% stripped FBS. Although we always completed our experiments within 24 hr, we observed a decrease in proliferation of H295R cell after 36–48 hr of co-culture with BeWo cells (see Supplemental Material, Figure S2C), suggesting that the presence of BeWo cells in the inserts affects the long-term proliferation of H295R cells in co-culture. This may be attributable to the far higher levels of total estrogens produced by BeWo cells in co-culture because greater concentrations of estradiol (10^–6^ M) are known to inhibit H295R cell proliferation (Jaroenporn et al. 2008). Various other hormones uniquely secreted by BeWo cells, such as β-hCG, could also be contributing to altered H295R cell proliferation because H295R cells are known to express functional LH (luteinizing hormone)/hCG receptors (Rao et al. 2004). Whether β-hCG affects fetal adrenal cell proliferation or function *in utero* remains to be investigated, but in the present study we used β-hCG secretion levels as an established indicator of normal trophoblast function to confirm that biochemical differentiation of BeWo cells occurred appropriately in our co-culture model. We observed a β-hCG secretion rate that was somewhat greater in co-culture medium than under regular culture conditions (see Supplemental Material, Table S2). This increase, evident after 48 hr, but no longer apparent after 72 hr, may have been attributable to the insulin in the co-culture medium because insulin is known to increase β-hCG production in villous trophoblast cells (Ren and Braunstein 1991).

*Steroidogenesis in the co-culture model*. Consistent with the functional steroidogenic fetoplacental unit during human pregnancy, our co-culture model is capable of progesterone, androgen, and estrogen biosynthesis *de novo* (Figure 1). In our co-culture, progesterone production occurs predominantly in BeWo cells, consistent with the progesterone biosynthetic function of the trophoblast (Braunstein 2003), which is essential for maintenance of pregnancy (Wetendorf and DeMayo 2012). Moreover, precursors for estrogens are produced *de novo* predominantly by H295R cells (DHEA and androstenedione, but no detectable testosterone).

CYP19 is expressed and active in H295R and BeWo cells, but different tissue-specific promoters are involved in its expression in each cell type (Klempan et al. 2011; Sanderson et al. 2004). In the fetoplacental unit, CYP19 is mainly regulated via the PKC pathway through the major placental I.1-promoter of *CYP19* (Harada et al. 2003). Although human fetal tissues possibly contain CYP19 activity and/or *CYP19* transcripts (Pezzi et al. 2003), CYP19 levels are low and its regulation is not understood. Fetal aromatase transcript appears to be mainly derived from the gonadal pII-promoter of *CYP19*, which is regulated by gonadotropins, including hCG, via the Gs-protein-coupled follicle stimulating hormone (FSH) and LH/hCG receptors that activate cyclic adenosine monophosphate (cAMP)/PKA signaling, suggesting that this pathway is involved in fetal CYP19 regulation (Bulun et al. 1994). Our co-culture system responded to stimulation of the PKC and PKA signaling pathways with increased CYP19 activity (see Supplemental Material, Figure S3). The relative contribution of basal or induced CYP19 activity was considerably (15 times) greater in BeWo than H295R cells (data not shown), which is consistent with evidence that *CYP19* gene expression and CYP19 catalytic activity are far greater in placental tissue than fetal adrenal or fetal liver tissue (Pezzi et al. 2003).

A relevant *in vitro* steroidogenic model of the fetoplacental unit requires the *de novo* production of estrogens, including the pregnancy-specific hormone estriol, which is an indicator of fetal well-being (Mucci et al. 2003). We found basal estrogen production by H295R and BeWo cells in monoculture to be very low; however, in co-culture, estradiol and estriol production increased synergistically. Estriol production is almost uniquely (> 90%) dependent on the fetal precursor 16α-hydroxyandrostenedione produced by fetal hepatic CYP3A7 (Kitada et al. 1987). The synergistic production of estriol by our co-culture indicates that H295R cells are also acting as a suitable (steroidogenic) model for the fetal liver by providing the 16α-hydroxyandrostenedione precursor. Estrone production during pregnancy is lower than that of estriol and estradiol, and its levels do not correlate with the other estrogens; neither is its function during pregnancy well understood (Braunstein 2003). Estrone was produced by H295R and BeWo cells in monoculture; in co-culture, estrone production was increased additively, not synergistically, which is again consistent with the observed kinetics of estrogens during human pregnancy (Tulchinsky et al. 1972).

Taken together, the steroidogenic profile of our co-culture model—given the lack of quantifiable testosterone production but great production of DHEA and, to a lesser extent, androstenedione—indicates that estradiol is produced mainly via the aromatization of androstenedione to estrone and its subsequent rapid conversion to estradiol by 17β-hydroxysteroid dehydrogenase type 1 (HSD17B1). Estradiol production would thus be achieved without the requirement for the synthesis of large quantities of the potent androgen testosterone, which could cause inappropriate masculizing/defeminizing of the fetus. This explanation is plausible because HSD17B1 is known to be highly expressed in BeWo cells (Lewintre et al. 1994), as it is in human trophoblast cells (Brown et al. 2003). Although H295R cells are known to express *HSD17B1* (Hilscherova et al. 2004), this isoform is not effective at converting androstenedione to testosterone (Poirier 2010). On the other hand, H295R cells express *HSD17B4*, which has dehydrogenase (oxidative) activity and would favor the conversion of testosterone to androstenedione (Poirier 2010). Although testosterone production in H295R cells has been reported, our inability to detect significant quantities of testosterone are consistent with the original studies that characterized the steroidogenic profile of H295R and its parent line NCI-H295 (Gazdar et al. 1990; Rainey et al. 1994).

*Disruption of fetoplacental steroidogenesis by endocrine-disrupting pesticides*. Atrazine is a member of the triazine herbicide family and is suspected to have long-term adverse environmental effects (Jablonowski et al. 2011). Adverse birth outcomes (fetal growth restriction and preterm birth) have been associated with atrazine exposure (Chevrier et al. 2011; Rinsky et al. 2012). The endocrine-disruptive effect of atrazine on CYP19 has been studied in several cell models (Fan et al. 2007; Sanderson et al. 2000). Atrazine induces *CYP19* expression in H295R cells via the I.3 and pII promoters by increasing the intracellular levels of cAMP (Sanderson et al. 2002). In addition, an interaction of atrazine with steroidogenic factor 1, a transcription factor required for activation of the pII promoter of *CYP19*, may be involved (Fan et al. 2007).

In our co-culture, atrazine increased CYP19 activity in H295R cells only (Figure 2), which is consistent with the importance of the PKA pathway in the pII promoter-driven regulation of *CYP19* in these cells, whereas in placental cells, *CYP19* is under the control of the PKC-responsive I.1 promoter (Watanabe and Nakajin 2004). Atrazine did not modify aromatase activity in BeWo cells, although forskolin induced CYP19 activity in this cell line. We suggest that forskolin increases aromatase activity indirectly—as a result of its known stimulatory effect on BeWo cell syncytialization, which is normally associated with increased CYP19 expression (Taylor et al. 1991). Atrazine does not have this effect and it did not affect β-hCG levels (data not shown). Despite the induction by atrazine of CYP19 activity in H295R cells (the fetal compartment), estradiol and estriol production by the co-culture (the cooperative fetoplacental unit) was not altered, indicating that the contribution of “fetal” CYP19 to overall estrogen production is small, if not negligible, in our co-culture model, as it is in the human fetoplacental unit *in vivo.* This furthermore emphasizes the relevance of the tissue-specific and condition-specific (pregnancy) nature of the regulation of CYP19 in humans. Chemicals that induce aromatase expression and activity in selected *in vitro* cell systems may have very different, if any, effects *in vivo* if the tissue-specific mechanisms of such observed induction is not taken into consideration.

Prochloraz, a fungicide with antiandrogenic properties, has a range of actions on cytochrome P450 enzymes (Vinggaard et al. 2002, 2005). Perinatal exposure of rats to prochloraz resulted in feminization of the male pups, which was associated with reduced testosterone levels, likely due to inhibition of CYP17 (Vinggaard et al. 2005). In rats exposed prenatally to prochloraz, malformations of the male reproductive tract were observed (Noriega et al. 2005). Our laboratory previously showed prochloraz to be a mixed-type catalytic inhibitor of CYP19 activity in H295R cells (Sanderson et al. 2002). However, in the present study we did not observe such inhibition in H295R cells in our co-culture (Figure 3). This discrepancy may be attributed to different experimental conditions, including our lower cell densities and the presence of 1% (stripped) FBS in the co-culture medium. Prochloraz clearly inhibited CYP19 activity in BeWo cells (Figure 3). Consistent with the behavior of the human fetoplacental unit in which estrogen production is predominantly dependent on placental CYP19, prochloraz reduced estrogen production by the co-culture to background levels despite its lack of inhibition of “fetal” aromatase. The observation that estrogen production by the co-culture was already decreased by 90% at a prochloraz concentration of 1 μM that only partially inhibited CYP19 activity in BeWo cells (and not at all in H295R cells) may be explained by the known inhibitory effect of prochloraz on CYP17 activity, which would reduce the essential supply of precursors androgens from the H295R cells. Indeed, prochloraz (1 μM) inhibited DHEA production in co-culture by 97% (data not shown). Because the estrogen receptor is involved in H295R cell proliferation and antiestrogens inhibit H295R cell proliferation (Montanaro et al. 2005), it is not surprising to observe a decreased proliferation of H295R but not BeWo cells in the co-culture treated with prochloraz. This endocrine-disrupting effect of prochloraz could affect pregnancy outcome because estrogen deprivation is associated with a spontaneous abortion rate of 50% in the baboon, a species commonly used as a model for primate/human pregnancy (Albrecht and Pepe 1999).

**Figure 4:**
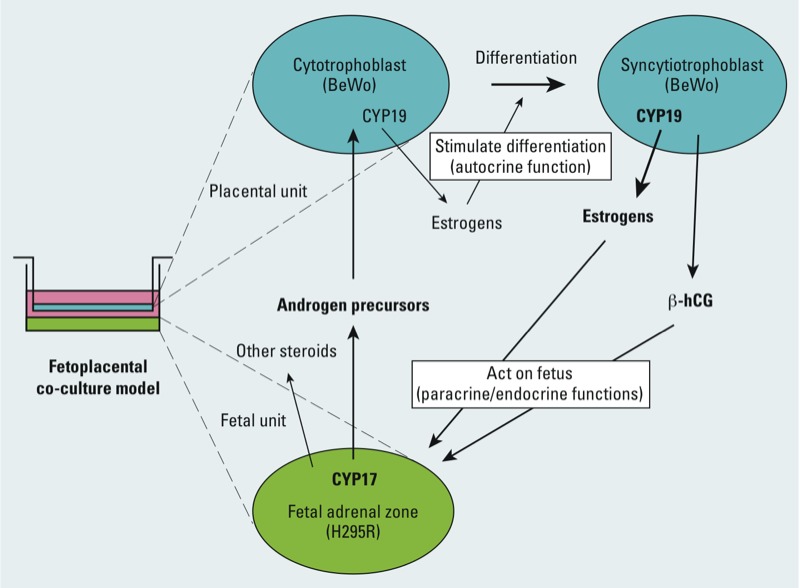
Schematic representation of the fetoplacental interactions in our co-culture model of H295R human (fetal-like) adrenocortical carcinoma and BeWo human (trophoblast-like) choriocarcinoma cells. The fetal unit expresses CYP17 (steroid 17α-hydroxylase/17,20-lyase) and produces androgen precursors, which are converted to estrogens by the placental aromatase (CYP19). Placental estrogens and β-hCG act in autocrine, paracrine, and endocrine manners on the trophoblast and fetal unit. Enzymes/hormones in bold type indicate relatively greater activities/levels.

*A new tool to study steroidogenesis*. We developed our model to respond to the demand for noninvasive *in vitro* research tools for studying the effects of chemical exposures during pregnancy on placental and fetal health. Our co-culture of BeWo and H295R cells not only allows the study of the complete fetoplacental steroidogenesis pathway, it also takes into consideration the impacts of numerous fetoplacental interactions (Figure 4), which occur in real time, affecting the behavior of both cell types. For instance, placental β-hCG appears to be involved in the regulation of DHEA sulfation via LH/hCG receptors present in the H295R cells by stimulating sulfotransferases (Rao et al. 2004) and could affect availability of androgen precursors for BeWo cells in the co-culture. Moreover, several steroid hormones produced in the placenta, such as estradiol and progesterone, also regulate the expression of the 11β-hydroxysteroid dehydrogenases (HSD11B), which play a role in the regulation of fetal growth and development of the fetal adrenal zone (Beaudoin et al. 1997; Kaludjerovic and Ward 2012; Myatt and Sun 2010). The ability of the co-culture to produce mineralo- and glucocorticoids also allows for the study of stress responses and homeostasis.

## Conclusions

The co-culture of H295R and BeWo cells is a unique *in vitro* model that reproduces the steroidogenic cooperation between the fetal adrenal/liver and the villous trophoblast during pregnancy. The model provides a versatile tool to study the impact of potential endocrine-disrupting chemicals (e.g., environmental contaminants, drugs) to which pregnant women may be exposed.

## Supplemental Material

(717 KB) PDF
