# Supplementary material for: A Unique Co-culture Model for Fundamental and Applied Studies of Human Fetoplacental Steroidogenesis and Interference by Environmental Chemicals
Source: Environ Health Perspect. 2014 Jan 31;122(4):371–7. doi: 10.1289/ehp.1307518 (PMC3984223; doi:10.1289/ehp.1307518)
Supplement: (717 KB) PDF [file ehp.1307518.s001.pdf]

Environmental Health Perspectives doi :10.1289/ehp.1307518

Supplemental Material

A Unique Co-culture Model for Fundamental and Applied Studies of Human Fetoplacental Steroidogenesis and Interference by Environmental Chemicals

Andrée-Anne Hudon Thibeault, Kathy Deroy, Cathy Vaillancourt, and J. Thomas Sanderson

Table of Contents **Page**

Figure S1. Experimental design of the co-culture experiments with description of the composition of the co-culture medium. **2**

Figure S2. H295R (A) and BeWo (B) cell proliferation in regular (ATCC-recommended) medium or in co-culture medium. H295R (C) and BeWo (D) cell proliferation in co-culture medium either as monocultures or in co-culture with the other cell line. **3**

Figure S3. Relative CYP19 activity in H295R (A) and BeWo (B) cells cultured in their respective regular media or in co-culture medium after 24 h exposure to formestane (1 µM), phorbol-12-myristate-13-acetate (PMA; 1 µM) or forskolin (10 µM). **4**

Table S1. Sensitivity of the ELISA kits used to detect and quantify the cellular production of β−hCG and steroid hormones. **5**

Table S2. Basal and forskolin-stimulated (10 µM) β-hCG production (mIU/mL) by BeWo cells in regular or in co-culture medium over a 24, 48 or 72 h period of monoculture or after 24 h in co-culture with H295R cells. **6**

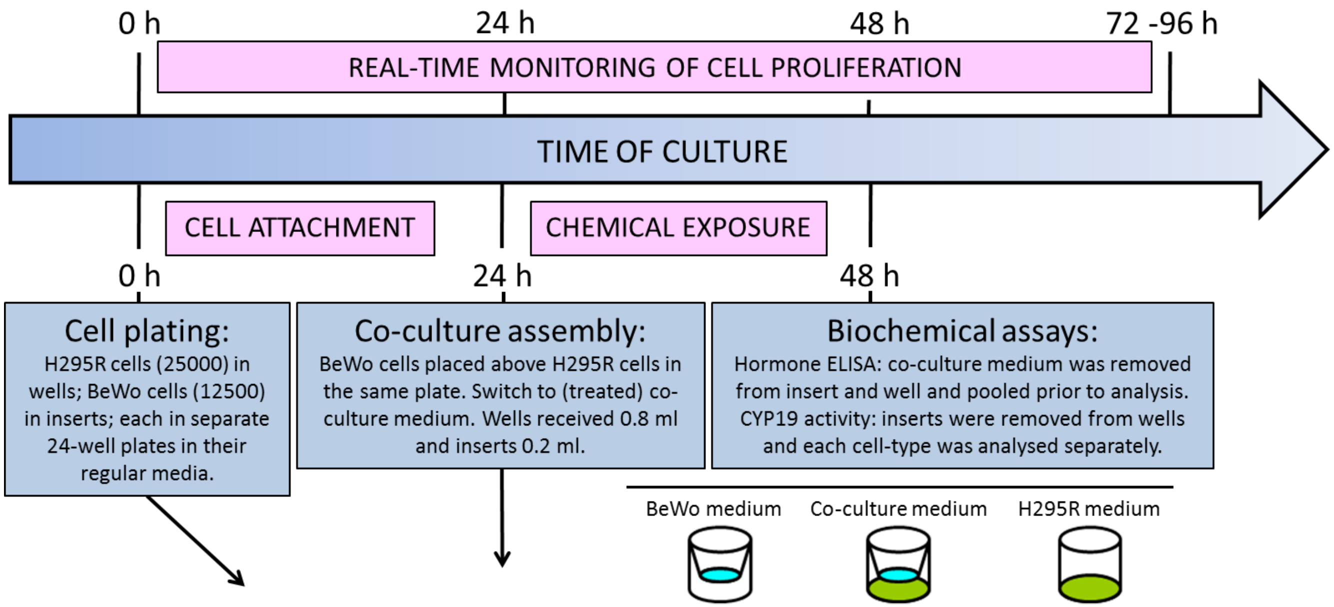

	BeWo medium	Co-culture medium	H295R medium
Culture medium	DMEM/F-12 HAM without phenol red		
$NaHCO_3$ (Sigma-Aldrich, D2906)	0.6 g/L	1.2 g/L	1.2 g/L
FBS (Hyclone, SH30070)	10 %	-	-
Charcoal-stripped FBS (Sigma-Aldrich, F6765)	-	1 %	-
Nuserum (BD Biosciences, 355500)	-	2.5 %	2.5 %
Pyridoxine•HCl (Sigma-Aldrich, P6280)	-	2 mg/L	2 mg/L
ITS + Premix (BD Bioscience, 354352) (Insulin 0.625 mg/mL; transferrin 0.625 mg/mL; selenous acid,0.625 µg/mL; BSA,0.125 g/ml; linoleic acid , 5.35 mg/mL)	-	1 %	1 %

Figure S1. Experimental design of the co-culture experiments with description of the composition of the co-culture medium.

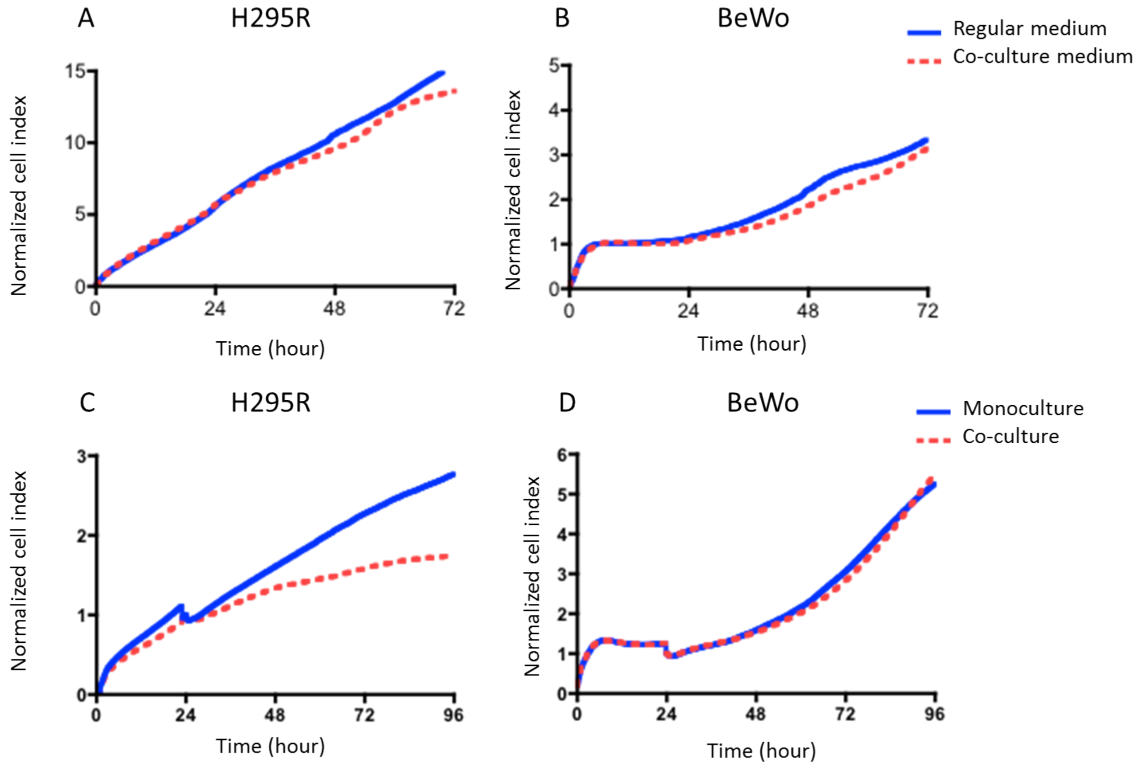

Figure S2. H295R **(A)** and BeWo (**B**) cell proliferation in regular (ATCC-recommended) medium or in co-culture medium. H295R **(C)** and BeWo (**D**) cell proliferation in co-culture medium either as monocultures or in co-culture with the other cell line. We monitored cell proliferation in real-time using an impedance-based xCELLigence™ RTCA DP instrument (ACEA Biosciences, San Diego, CA). We normalized cell index after complete adhesion of H295R (at 3 h, **A**) or BeWo (at 6 h, **B**) cells or, in the case of co-culture, 24 h after plating and immediately after co-culture assembly (**C, D**). Each trace is the average of three measurements.

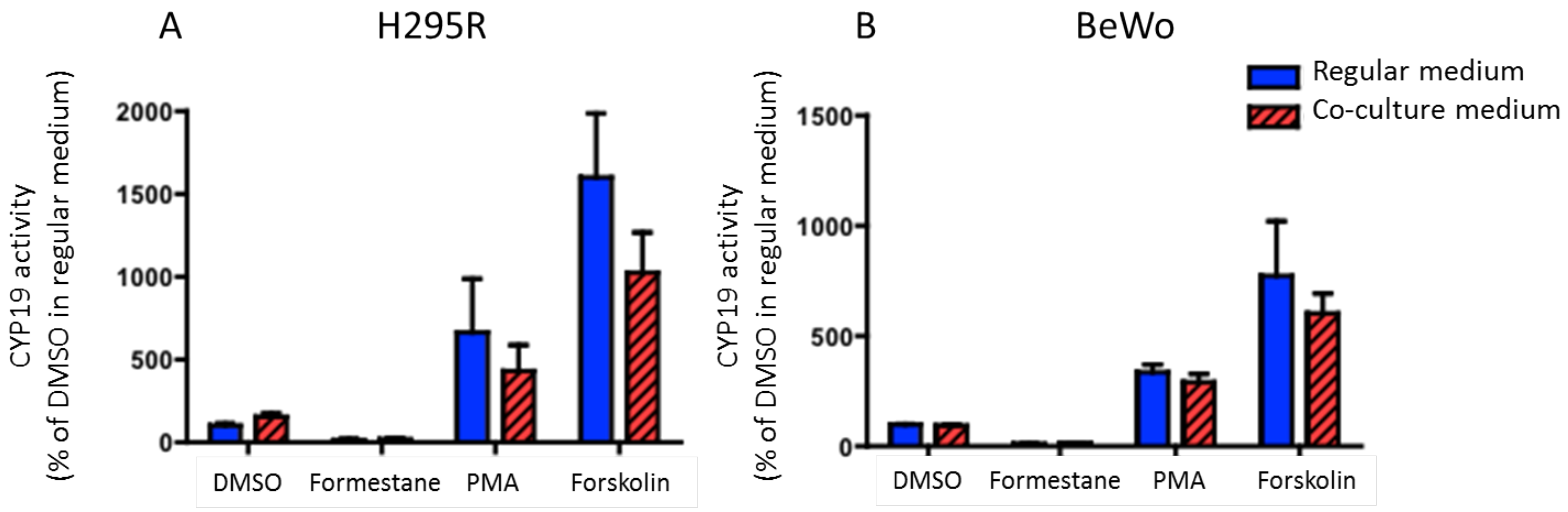

Figure S3. Relative CYP19 activity in H295R (**A**) and BeWo (**B**) cells cultured in their respective regular media or in co-culture medium after a 24 h exposure to formestane (1 µM), phorbol-12-myristate-13-acetate (PMA; 1 µM) or forskolin (10 µM). Activities were expressed as percentage (mean ± SEM; $n = 3$) of the activities in cells exposed to vehicle control (DMSO; 0.1% v/v) in their respective regular culture media. Two-way ANOVA did not detect a statistically significant ($P < 0.05$) effect of the co-culture medium on basal or induced CYP19 activities. Regardless of medium, inducibility by PMA ($P < 0.05$) and forskolin ($P < 0.001$) was statistically significantly greater in H295R than in BeWo cells.

Table S1. Sensitivity of the ELISA kits used to detect and quantify the cellular production of β−hCG and steroid hormones.

**Hormone**	**ELISA kit Company, catalogue number**	**Sensitivity of the kit (β−hCG: mIU/mL) (steroids: pg/mL)**	**Lowest concentration detected in cell culture (basal 24 h production) (β−hCG: mIU/mL) (steroids: pg/mL)**
β−hCG	DRG Diagnostics EIA-1911	1.0	7.7 ± 1.8 [a]
Progesterone	DRG Diagnostics EIA-1561	45	1697 ± 257
Dehydroepiandrosterone (DHEA)	DRG Diagnostics EIA-3415	108	< 108 [b]
Androstenedione	DRG Diagnostics EIA-3265	19	< 19 [b]
Testosterone	DRG Diagnostics EIA-1559	83	≤ 83 [b, c]
Estradiol	DRG Diagnostics EIA-2693	9.7	11.0 ± 2.0
Estriol	DRG Diagnostics EIA-3717	40	(35 ± 35) [c]
Estrone	Abnova KA-1908	10.0	11.7 ± 3.2

[a]β-hCG was detectable in BeWo cells only. [b]Levels were below the limit of detection in BeWo cells. [c]Levels were at or below the limit of detection in H295R cells.

Table S2. Basal and forskolin-stimulated (10 µM) β-hCG production (mIU/mL) by BeWo cells in regular or in co-culture medium over a 24, 48 or 72 h period of monoculture or after 24 h in co-culture with H295R cells.

Treatment	Regular medium	Co-culture medium	In co-culture with H295R cells
**Basal**			
24 h	7.7 ± 1.8	10.9 ± 3.2	36.0 ± 8.7[a]
48 h	40.5 ± 9.3	80.5 ± 17.8*	-
72 h	89.1 ± 14.1	88.5 ± 19.6	-
**Forskolin**			
24 h	156.7 ± 93.5	177.4 ± 18.1	241.8 ± 41.8[a]
48 h	1126.7 ± 409.7	3626.1 ± 444.7*	-
72 h	4918.1 ± 2178.3	4952.1 ± 617.7	-

*Statistically significant difference from corresponding production in regular medium over the same period determined by two-way ANOVA ($p < 0.05$) and Bonferroni post-hoc test.

[a]Note that in co-culture the exposure regime was as described in Supplemental Material, Figure S1 and is not directly comparable to the β-hCG production levels in monoculture.
